# Supplementary material for: Nutritional status and effective verbal communication in Peruvian children: A secondary analysis of the 2019 Demographic and Health Survey
Source: PLoS One. 2021 Feb 18;16(2):e0246542. doi: 10.1371/journal.pone.0246542 (PMC7891687; doi:10.1371/journal.pone.0246542)
Supplement: Attachment — Submitted filename: PONE-D-20-26209_reviewer.pdf [file pone.0246542.s003.pdf]

PLOS ONE

Impact of nutritional status on effective verbal communication in Peruvian children. A nationwide study in Peru

--Manuscript Draft--

**Manuscript Number:**	PONE-D-20-26209
**Article Type:**	Research Article
**Full Title:**	Impact of nutritional status on effective verbal communication in Peruvian children. A nationwide study in Peru
**Short Title:**	Impact of nutritional status on effective verbal communication in Peruvian children
**Corresponding Author:**	Rodrigo Vargas-Fernández, M.D.<br>Universidad Cientifica del Sur<br>Lima, Peru PERU
**Keywords:**	Child; Child, Preschool; Humans; Prevalence; Propensity Score; Cross-Sectional Studies; Surveys and Questionnaires; Communication; Child Development; Latin America; Malnutrition; Peru
**Abstract:**	Background<br><br>To estimate the impact of chronic malnutrition on the development of effective verbal communication (EVC) in children aged 24 to 36 months.<br><br>Methods<br><br>We conducted a retrospective, quasi-experimental study design using data from 4452 children between 24 and 36 months of age available in the Early Childhood Development (ECD) section of the ENDES 2019 survey. Achievement of ECV was considered as the dependent variable. After a propensity score matching analysis, a total 601 children were included in the exposed (children with chronic malnutrition) and 3848 in the unexposed group (children without chronic malnutrition). The psmatch2 Stata software command was used to estimate the impact of chronic malnutrition on ECV with a level of 5% for significance.<br><br>Results<br><br>The EVC indicator was achieved by 57.4% of the children between 24 and 36 months of age evaluated, while the prevalence of chronic malnutrition in this population was 14%. The evaluation of impact showed that the group exposed to chronic malnutrition was 8% less likely to show ECV compared to the unexposed group (ATT -0.08; 95% CI -0.106 to -0.054; p<0.001).<br><br>Conclusions<br><br>The presence of chronic malnutrition was found to have a significant impact on the propensity to show EVC in Peruvian children between 24 and 36 months of age. Strengthening strategies for reducing malnutrition in vulnerable areas, as well as those directly affecting ECV is a priority for diminishing gaps in the achievement of this indicator in our country.
**Order of Authors:**	Akram Hernández-Vásquez
	Nilthon Pisfil-Benites
	Rodrigo Vargas-Fernández
	Diego Azañedo
**Additional Information:**	
**Question**	**Response**
**Financial Disclosure**	The authors received no specific funding for this work.

Powered by Editorial Manager® and ProduXion Manager® from Aries Systems Corporation

Enter a financial disclosure statement that describes the sources of funding for the work included in this submission. Review the submission guidelines for detailed requirements. View published research articles from *PLOS ONE* for specific examples.<br><br>This statement is required for submission and **will appear in the published article** if the submission is accepted. Please make sure it is accurate.<br><br>**Unfunded studies**<br>Enter: *The author(s) received no specific funding for this work.*<br><br>**Funded studies**<br>Enter a statement with the following details:<br>• Initials of the authors who received each award<br>• Grant numbers awarded to each author<br>• The full name of each funder<br>• URL of each funder website<br>• Did the sponsors or funders play any role in the study design, data collection and analysis, decision to publish, or preparation of the manuscript?<br>• **NO** - Include this sentence at the end of your statement: *The funders had no role in study design, data collection and analysis, decision to publish, or preparation of the manuscript.*<br>• **YES** - Specify the role(s) played.<br><br>* typeset	
**Competing Interests**<br><br>Use the instructions below to enter a competing interest statement for this submission. On behalf of all authors, disclose any competing interests that could be perceived to bias this work—acknowledging all financial support and any other relevant financial or non-financial competing interests.<br><br>This statement **will appear in the published article** if the submission is accepted. Please make sure it is accurate. View published research articles	The authors have declared that no competing interests exist.

from *PLOS ONE* for specific examples.<br><br>**NO authors have competing interests**<br><br>Enter: *The authors have declared that no competing interests exist.*<br><br>**Authors with competing interests**<br><br>Enter competing interest details beginning with this statement:<br><br>*I have read the journal's policy and the authors of this manuscript have the following competing interests: [insert competing interests here]*<br><br>* typeset	
**Ethics Statement**<br><br>Enter an ethics statement for this submission. This statement is required if the study involved:<br><br>• Human participants<br>• Human specimens or tissue<br>• Vertebrate animals or cephalopods<br>• Vertebrate embryos or tissues<br>• Field research<br><br>Write "N/A" if the submission does not require an ethics statement.<br><br>General guidance is provided below. Consult the submission guidelines for detailed instructions. **Make sure that all information entered here is included in the Methods section of the manuscript.**	The study did not require the approval of an ethics committee because it is an analysis of secondary data that is in the public domain and does not allow the identification of the participants evaluated.

Powered by Editorial Manager® and ProduXion Manager® from Aries Systems Corporation

**Format for specific study types**<br><br>**Human Subject Research (involving human participants and/or tissue)**<br>• Give the name of the institutional review board or ethics committee that approved the study<br>• Include the approval number and/or a statement indicating approval of this research<br>• Indicate the form of consent obtained (written/oral) or the reason that consent was not obtained (e.g. the data were analyzed anonymously)<br><br>**Animal Research (involving vertebrate animals, embryos or tissues)**<br>• Provide the name of the Institutional Animal Care and Use Committee (IACUC) or other relevant ethics board that reviewed the study protocol, and indicate whether they approved this research or granted a formal waiver of ethical approval<br>• Include an approval number if one was obtained<br>• If the study involved *non-human primates*, add *additional details* about animal welfare and steps taken to ameliorate suffering<br>• If anesthesia, euthanasia, or any kind of animal sacrifice is part of the study, include briefly which substances and/or methods were applied<br><br>**Field Research**<br><br>Include the following details if this study involves the collection of plant, animal, or other materials from a natural setting:<br>• Field permit number<br>• Name of the institution or relevant body that granted permission	
**Data Availability**<br><br>Authors are required to make all data underlying the findings described fully available, without restriction, and from the time of publication. PLOS allows rare exceptions to address legal and ethical concerns. See the PLOS Data Policy and FAQ for detailed information.	Yes - all data are fully available without restriction

A Data Availability Statement describing where the data can be found is required at submission. Your answers to this question constitute the Data Availability Statement and **will be published in the article**, if accepted.<br><br>**Important:** Stating 'data available on request from the author' is not sufficient. If your data are only available upon request, select 'No' for the first question and explain your exceptional situation in the text box.<br><br>Do the authors confirm that all data underlying the findings described in their manuscript are fully available without restriction?	
**Describe where the data may be found in full sentences. If you are copying our sample text, replace any instances of XXX with the appropriate details.**<br><br>• If the data are **held or will be held in a public repository**, include URLs, accession numbers or DOIs. If this information will only be available after acceptance, indicate this by ticking the box below. For example: *All XXX files are available from the XXX database (accession number(s) XXX, XXX.).*<br>• If the data are all contained **within the manuscript and/or Supporting Information files**, enter the following: *All relevant data are within the manuscript and its Supporting Information files.*<br>• If neither of these applies but you are able to provide **details of access elsewhere**, with or without limitations, please do so. For example:<br><br>*Data cannot be shared publicly because of [XXX]. Data are available from the XXX Institutional Data Access / Ethics Committee (contact via XXX) for researchers who meet the criteria for access to confidential data.*<br><br>*The data underlying the results presented in the study are available from (include the name of the third party*	All relevant data are within the manuscript and its Supporting Information files.

and contact information or URL).<br>• This text is appropriate if the data are owned by a third party and authors do not have permission to share the data.<br><br>* typeset	
Additional data availability information:	

Powered by Editorial Manager® and ProduXion Manager® from Aries Systems Corporation

Manuscript
Click here to access/download;Manuscript;Draft_Desnutricion_DIT_2008202

Impact of nutritional status on effective verbal communication in Peruvian children. A nationwide study in Peru

Akram Hernández-Vásquez[1¶], Nilthon Pisfil-Benites[2&], Rodrigo Vargas-Fernández[3#*], Diego Azañedo[4#]

[1] Centro de Excelencia en Investigaciones Económicas y Sociales, Vicerrectorado de Investigación, Universidad San Ignacio de Loyola, Lima, Peru

[2] Facultad de Economía y Planificación, Universidad Nacional Agraria de la Molina, Lima, Peru

[3] Universidad Científica del Sur, Lima, Peru

[4] Instituto de Investigación, Universidad Católica los Ángeles de Chimbote, Chimbote, Peru

* Corresponding author

Email: 100006379@ucientifica.edu.pe

[¶] Conceptualization, Data curation, Formal analysis, Methodology, Validation, Supervision, Writing – original draft, Writing – review & editing

[&] Formal analysis, Methodology, Validation, Writing – original draft, Writing – review & editing

[#] Validation, Writing – original draft, Writing – review & editing

Abstract

Background

To estimate the impact of chronic malnutrition on the development of effective verbal communication (EVC) in children aged 24 to 36 months.

Methods

We conducted a retrospective, quasi-experimental study design using data from 4452 children between 24 and 36 months of age available in the Early Childhood Development (ECD) section of the ENDES 2019 survey. Achievement of ECV was considered as the dependent variable. After a propensity score matching analysis, a total 601 children were included in the exposed (children with chronic malnutrition) and 3848 in the unexposed group (children without chronic malnutrition). The *psmatch2* Stata software command was used to estimate the impact of chronic malnutrition on ECV with a level of 5% for significance.

Results

The EVC indicator was achieved by 57.4% of the children between 24 and 36 months of age evaluated, while the prevalence of chronic malnutrition in this population was 14%. The evaluation of impact showed that the group exposed to chronic malnutrition was 8% less likely to show ECV compared to the unexposed group (ATT -0.08; 95% CI -0.106 to -0.054; p<0.001).

Conclusions

The presence of chronic malnutrition was found to have a significant impact on the propensity to show EVC in Peruvian children between 24 and 36 months of age. Strengthening strategies for reducing malnutrition in vulnerable areas, as well as those directly affecting ECV is a priority for diminishing gaps in the achievement of this indicator in our country.

Introduction

Child malnutrition produces serious metabolic and structural consequences for the individual and has a high social and economic impact [1], mainly in low- and middle-income countries, where nearly half of the children under five years of age die due to malnutrition [2]. The chronicity of this condition affects a large proportion of children under five throughout the world, with an estimated 23% (144 million) of people in this age group presenting stunting in 2019 [3]. In 2011, low- and middle-income countries reported the highest proportions of malnutrition with 28% of stunting in children, in comparison to the 7.2% reported in high-income countries [4]. Also in 2017, Peru reached a 12.9% of stunting in children under five years of age, which is under the average prevalence of 25% for that outcome in developing countries the same year [5]. This becomes relevant because children integral development at an early stage (perceptual, motor, cognitive, language, socio-emotional and self-regulation skills), is highly dependent of an active and healthy life, which cannot be well achieved in the presence of malnutrition [6].

Early childhood development (ECD) is one of the goals of the Sustainable Development Goals in developing countries [7]. However, in these countries, nearly 150 million children under five years of age are at risk of failing to reach their potential development due to malnutrition and extreme poverty [8]. ECD is essential to achieve adequate learning, occupational productivity, physical and psychological health, and social well-being [9]. In Peru, the government, led by the Ministry of Development and Social Inclusion, implemented a program aimed at improving ECD. This program included seven areas of development corresponding to a healthy birth, safe attachment, adequate nutritional status, effective verbal communication, walking alone, regulation of emotions and behaviors and symbolic function [8,10].

Effective verbal communication (EVC) begins to develop at an early age, through crying or other forms of rudimentary communication as an immediate mechanism of interrelationship between children and parents. This communication influences adolescent development in the academic stage and work productivity as well as the development of emotions in adult life [11]. To achieve adequate EVC, the nutrition of a child from the prenatal period through the first 24 months of life must be adequate because the risk of stunting during this period in developing countries is high, leading to consequences

in cognitive development [11]. Chronic malnutrition also creates learning difficulties in the first one thousand days of life and has an impact on EVC in the long term [12,13]. Therefore, in order to achieve the proper development of EVC, adequate nutrition and eating habits of the child must be ensured (during the first months of life) and those of the mother must be improved. Moreover, safe attachment of children and their parents is necessary as well as stimulation of motor developmental and gesticulation [14–16].

In Peru, there are no population-based studies on the impact of nutritional status on the development of EVC during childhood. However, the National Institute of Statistics and Informatics (INEI in Spanish) collects nutritional information on children under five years of age through the Demographic and Family Health Survey (ENDES in Spanish). During a recent validation of the ECD instrument the ENDES also included information on seven components of ECD including EVC [17]. Therefore, the objective of the present study was to estimate the effect of chronic malnutrition in children aged 24 to 36 months on the development of EVC.

Materials and methods

Design and study population

The ENDES is a survey representative of the Peruvian population. In a secondary analysis of the ENDES 2019, the sample was characterized by being bi-stage, probabilistic of a balanced, stratified and independent type, at the departmental level and by urban and rural areas. The sample size of ENDES 2019 was 36,760 homes, 9320 of which were in urban areas and 12,660 were from rural areas.

The study had a retrospective, quasi-experimental design and included 4452 children between 24 and 36 months of age. The children were analyzed in the ECD section of the ENDES 2019, which evaluates different aspects of child development including EVC. To estimate the impact of chronic child malnutrition on EVC the sample was divided into exposed (children with chronic malnutrition) and unexposed children (without chronic malnutrition).

Variables

The World Health Organization (WHO) classification was used for the exposure variable "malnutrition" (HW70) [18] and EVC in children aged between 24 to 36 months (R4). The latter variable, measures EVC behaviors at the comprehensive and expressive level for each evolutionary stage of children aged 24 to 36 months according to a psychological instrument (Battelle Development Instrument) previously validated by experts who agreed on the questions to be included by the calculation of an Aiken indicator V greater than 0.7 (85.2%). This instrument was previously used in a pilot test in four Peruvian cities to determine adequate functioning and is based on questions that are performed and answered by the mothers [19].

The study covariates (codes in parentheses) included: age of the child in months (QI478), age of the mother (V012), years of education of the mother (V133), hemoglobin level of children (HW53), sex of the child (B4), total householders (HV009), health insurance coverage (V481), total children ever born (V201) and cesarean delivery (M17). In addition, the following variables: were constructed: "quintile" of the wealth index (V190) categorized into five levels (poorest, poor, middle, rich, richest), "prenatal control" according to prenatal visits (inadequate [less than 8] and suitable [8 or more]) during pregnancy (M14), "altitude" (m. a. s. l.) of the place of residence (HV040), "area" classifying the type of place of residence as urban or rural (V025), "ethnicity" (V131) considering ethnic origin (native and non-native), "natural region" including the natural region of origin (coast, highlands and jungle), "immediate lactation" considered as whether the infant was breastfed within the first hour after birth or not (V426), and "breastfeeding in months" according to the number of months the child was breastfed (M5). The covariates were selected according to revision of the literature available [20–23].

Statistical analysis

The model proposed by Roy-Rubin [24,25], was followed, defined as an exposure variable to chronic malnutrition (*Di*) and as an EVC outcome variable in children between 24 and 36 months (Y_i). That is, $Y_i(1)$ is the result variable if the individual *i* was exposed and $Y_i(0)$ is the result variable if the individual *i* was not exposed. Thus, the effect of exposure (τ_i) for an individual *i* can be calculated as:

$$\tau_i = Y_i(1) - Y_i(0)$$

The analysis focused on determining the average impact of exposure (Average Treatment Effect [ATE]) and the average treatment effect on the exposed population (Average Treatment on the Treated [ATT]).

$$\tau_{ATE} = E[Y_i(1) - Y_i(0)]$$

$$\tau_{ATT} = E[Y_i(1)|D_i = 1] - E[Y_i(0)|D_i = 1]$$

Where: $E[\,.\,]$ corresponds to an expectation operator.

In this case, the average impact of exposure in exposed subjects (τ_{ATT}) is the expected value of the difference in the result variable in the group of individuals exposed when there is the presence of exposure $E[Y_i(1)|D_i = 1]$ and the expected value of the result variable in the exposure group in the absence of exposure, known as contrafactual $E[Y_i(0)|D_i = 1]$. That is, the estimated impact is the difference in the average of the result variable of the exposure group and the control group.

The estimated impact is composed of the effect of exposure and selection bias. Formally, it is assumed that the selection bias is due only to differences in observable characteristics (conditional independence condition (IC)). What this assumption makes is to ensure that the selection bias is equal to zero, generating an unbiased estimate of the true effect of exposure. This is achieved by finding an individual exposed to malnutrition with the same observable characteristics in the control group.

An individual is an adequate control of an individual exposed to malnutrition, if both individuals are similarly likely to be exposed to malnutrition. This implies that the propensity score matching (PSM) method proposed by Rosenbaum Rubin (1983) can only be calculated in the Common Support Region (CS) to ensure that the exposure and control groups are very similar.

The PSM estimator is the average difference of the result variables of the individuals in the exposure group and the control group in the common support with an appropriate probability of exposure participation.

Propensity and match estimation

A logit model estimated the exposure probabilities for the covariates, i.e. the probability of participation with the observable characteristics of individuals. Having the probability of exposure and with the intention to compare the exposure impact results, a matching procedure was implemented with the kernel estimator that matches each respondent in the exposure group with a weighted average of all respondents in the control group. In addition, a matching procedure was estimated with the nearest neighbor, without replacement, using the estimated exposure probabilities, implementing a 1:1 correspondence structure. To improve the quality of the matching the condition that the maximum absolute difference between the exposure probabilities of matched respondents was not greater than 0.1 was imposed. Finally, an IPWRA matching procedure was implemented using weighted regression coefficients to calculate the impact of exposure, where weights are the estimated inverse probabilities of exposure.

Post-match balancing diagnosis

To ensure the balance of coincidence matching in the probability of exposure between the specified covariate distributions of the two exposure groups, two assumptions were required for correct estimation of the impact of exposure: a) the difference in the average of the exposure variable of the exposure and control group is composed of the effect of chronic malnutrition and the selection bias, and b) any combination of characteristics observed in the exposure group also exists in the control group, ensuring that the exposure and control groups are very similar or are within a common support. Compliance with these assumptions is assumed for the estimation of the PSM.

Post-matching, the covariate distributions between the exposure groups in the matching sample were evaluated using the standardized absolute mean differences (bias) and variance ratios. After correspondence, standardized differences in the means of the covariates are expected to be close to zero, and variance ratios are expected to be close to one, if there is an adequate balance of covariates.

Estimating the impact of chronic malnutrition on outcomes

The impact of chronic malnutrition on children between 24 and 36 months of age was estimated by comparing the results between those exposed and unexposed in a subsample of individuals with similar characteristics. To do this, the *psmatch2* command was used. In the case of the PSM estimate

per Kernel and the PSM estimate per nearest neighbor, the *teffectsipwra* command of Stata was also used for weighted effect estimates with adjusted regression, considering that all impact estimates were evaluated at the 5% significance level.

Sensitivity analysis

A sensitivity analysis was performed on the effect estimates using the limit approach of Rosenbaum. This approach explains that if there were the influence of unmeasured covariates, then a match between the two groups of covariates would not have the same probability of exposure allocation, and their probability ratio (Γ) would be different from one. By increasing the value of Γ to identify the probabilities of exposure allocation (Γ>1), the degree of influence that an unmeasured covariate must have on the impact allocation and the validity of a study can be observed. Sensitivity analyses were performed for different values of Γ>1, in increments of 0.05, to determine the extent to which the Γ value remained at a significance level of 0.05. The inferences are then considered sensitive to bias caused by non-measured covariates if the Γ values are closer to 1 by altering the results (>0.05), and are considered relatively robust if values greater than Γ are required to obtain results that affect inference. The *mhbounds* command of Stata was used for this sensitivity analysis [24].

Ethical considerations

The study did not require the approval of an ethics committee because it is an analysis of secondary data that is in the public domain and does not allow the identification of the participants evaluated.

Results

Table 1 shows the characteristics of the 4452 children included, of which 604 (13.6%) were exposed to chronic malnutrition (exposure group) and 3848 (86.4%) were not exposed (control group). The mean current age of the mother was 30 years, while the mean lactation period of the child was 19 months, and the mean hemoglobin level of the child was 12.2 g/dl. In addition, the highest proportion in the wealth index was the poor (28.3%) and very poor quintiles (26.1%); 27.8% were from rural areas,

42.2% were from the geographical area of the Coast; 82.6% had health insurance; 57.4% of the children evaluated reached the EVC indicator.

Table 1. Characteristics of the study population (n=4452).

Characteristics	n=4452
**Current age in months, mean (sd)**	30.0 (3.7)
**Current age of mother in years, mean (sd)**	30.2 (6.8)
**Mother years education, mean (sd)**	10.5 (3.8)
**Altitude of the place of residence in meters, mean (sd)**	1269.0 (1420.1)
**Months of breastfeeding, mean (sd)**	19.1 (8.0)
**Household size, mean (sd)**	5.1 (2.0)
**Hemoglobin level of children, mean (sd)**	12.2 (1.3)
**Children ever born, mean (sd)**	2.4 (1.5)
**Q0 (richest), n (%)**	484 (10.9)
**Q1, n (%)**	662 (14.9)
**Q2, n (%)**	883 (19.8)
**Q3, n (%)**	1260 (28.3)
**Q4 (poorest), n (%)**	1163 (26.1)
**Prenatal control, n (%)**	2984 (67.0)
**Male children, n (%)**	2256 (50.7)
**Rural residence, n (%)**	1238 (27.8)
**Coast, n (%)**	1878 (42.2)
**Highlands, n (%)**	1466 (32.9)
**Jungle, n (%)**	1108 (24.9)
**Immediate lactation, n (%)**	2342 (52.6)
**Native ethnicity, n (%)**	390 (8.8)
**Health insurance**	3679 (82.6)
**Cesarean delivery, n (%)**	1428 (32.1)

Table 2 shows the distribution and comparison of covariates between the exposed and unexposed groups in matched and unmatched samples, of which four covariates had biases greater than +/- 0.75 after matching, while 10 covariates had differences +/- 0.25 in lower matching biases. Overall, bias of the model decreased from 31.1 to 2.0 after matching. Among exposed children, the altitude of the conglomerate, hemoglobin levels, live births and ethnicity were positively associated with exposure (0.01 - 0.03), while the current age of the mother, months of lactation, higher wealth quintile and residents in the jungle region (0.80-0.98) were less likely to be exposed.

Table 2. Comparison of background covariates between exposure groups in the unmatched and matched sample.

Characteristics	Unmatched				Matched			
	Exposure	Control	Difference		Exposure	Control	Difference	
	(n=604)	(n=3848)	t-stat	P-value	(n=601)	(n=3848)	t-stat	P-value
**Current age in months, mean (sd)**	30.018	30.026	-0.05	0.962	30.005	30.059	-0.25	0.800
**Current age of mothers in years, mean (sd)**	29.344	30.294	-3.19	0.001	29.313	29.658	-0.84	0.399
**Mother years education, mean (sd)**	8.066	10.914	-17.64	<0.001	8.102	8.252	-0.66	0.509
**Altitude of the place of residence in meters, mean (sd)**	1950.400	1162.000	12.92	<0.001	1944.500	1946.100	-0.02	0.986
**Months of breastfeeding, mean (sd)**	18.745	19.167	-1.21	0.227	18.772	19.189	-0.98	0.325
**Household size, mean (sd)**	5.545	5.026	6.03	<0.001	5.531	5.497	0.27	0.791
**Hemoglobin level of children, mean (sd)**	12.462	12.163	5.08	<0.001	12.463	12.457	0.07	0.943
**Children ever born, mean (sd)**	3.073	2.350	11.12	<0.001	3.052	3.052	-0.01	0.994
**Q0 (richest), n (%)**	0.028	0.121	-6.88	<0.001	0.028	0.038	-0.91	0.364
**Q1, n (%)**	0.053	0.164	-7.15	<0.001	0.053	0.058	-0.34	0.730
**Q2, n (%)**	0.083	0.216	-7.71	<0.001	0.083	0.088	-0.29	0.771
**Q3, n (%)**	0.253	0.288	-1.74	0.081	0.255	0.243	0.46	0.648
**Q4 (poorest), n (%)**	Ref.	-	-	-	Ref.	-	-	-
**Prenatal control, n (%)**	0.556	0.688	-6.44	<0.001	0.559	0.572	-0.45	0.650
**Children men, n (%)**	0.531	0.503	1.31	0.191	0.531	0.534	-0.09	0.925
**Rural residence, n (%)**	0.565	0.233	17.47	<0.001	0.562	0.550	0.44	0.660
**Coast, n (%)**	Ref.	-	-	-	Ref.	-	-	-
**Highlands, n (%)**	0.541	0.296	12.12	<0.001	0.539	0.543	-0.13	0.896
**Jungle, n (%)**	0.280	0.244	1.89	0.059	0.281	0.260	0.81	0.418
**Immediate lactation, n (%)**	0.652	0.506	6.72	<0.001	0.652	0.652	0	0.998
**Native ethnicity, n (%)**	0.219	0.067	12.45	<0.001	0.216	0.216	0.03	0.979
**Health insurance, n (%)**	0.894	0.816	4.73	<0.001	0.894	0.891	0.16	0.875
**Cesarean delivery, n (%)**	0.185	0.342	-7.71	<0.001	0.186	0.196	-0.43	0.668

**Mean standardized bias**		31.1				2.0		
**Pseudo R2 (logit)**		0.163				0.002		

As a result of the matching technique, all children found their unexposed counterpart, and the coincidence eliminated three observations. After matching, all covariates showed standardized means close to zero. The propensity score was estimated for each of the observations, and the balance was diagnosed, verifying the quality of the matching and imposing the common support. Fig 1 shows the assessment of the assumption of overlap.

Figure 1. Propensity score histogram.

Estimation of exposure impact

The estimate of the impact of chronic malnutrition is presented in Table 3. By way of comparison, the results of three different PSM estimators are presented. The estimated PSM Kernel shows the impact of chronic malnutrition on children between 24 and 36 months of age, which indicates that the group exposed to chronic malnutrition were 8% less likely to show EVC compared to the unexposed group, with a 95% confidence interval of -0.106 to -0.054 (95% CI). The nearest per-neighbor estimator (noreplacement) and weighting with adjusted regression (IPWRA) showed similar results of 9% (IC -0.144 to -0.034) and 6.3% (IC -0.119 to -0.005), respectively. After matching, the covariates in the two groups the mean standardized biases decreased from 31.1 to 2.0 (Fig 2), of which 15 observed variables were in the range of [-0.05,+0.05] after matching, and 5 observed variables showed a higher matching to the aforementioned range. The matching bias in all covariates was less than [- 0.98;+ 0.81].

Table 3. Exposure effects of chronic malnutritrion on EVC.

Variable	Exposure	Control	ATT	t test	SE	Z	p value	IC 95%
**Kernel**	0.489	0.569	-0.080	-3.31	0.024	-6.00	<0.001	-0.106 a -0.054
**Noreplacement (0.1)**	0.489	0.579	-0.09	-3.07	0.029	-3.20	<0.001	-0.144 a -0.034
**Ipwra**			0.063		0.03	-2.16	<0.031	-0.119 a -0.005

ATT: Average Treatment on the Treated.
SE: standard error.

Figure 2. Covariates balance before and after matching.

Sensitivity analysis

Tabla 4 shows a sensitivity analysis of Rosenbaum limits for estimating the impact of chronic malnutrition on EVC in children aged 24 to 36 months. Under the assumption that there is no influence of non-observable (non-measured) variables, the rate of possibility (Γ) was 1 and the estimate of the effect was significant. If there were factors that had not been considered in the modeling that affect the response variable, chronic malnutrition in children aged 24 to 36 months was less likely to have EVC, then the current estimate would have minimized the effects of exposure. Under the assumption of underestimation, a seamless variable would have to influence the odds exposure ratio to differ between the two exposure groups by a factor of more than 1.2 so that the estimate is not significant at the 5% level. However, assuming an overestimation, the estimate remained significant at all levels considered to be Γ suggesting that it was unlikely to be overestimated.

Table 4. Sensitivity analysis.

Gamma Γ	p_mh+	p_mh-
**1.000**	0.000	0.001
**1.050**	0.000	0.005
**1.100**	0.000	0.014
**1.150**	0.000	0.035
**1.200**	0.000	0.073
**1.250**	0.000	0.133
**1.300**	0.000	0.218
**1.350**	0.000	0.323
**1.400**	0.000	0.440
**1.450**	0.000	0.489
**1.500**	0.000	0.377
**1.550**	0.000	0.277
**1.600**	0.000	0.195
**1.650**	0.000	0.131
**1.700**	0.000	0.085
**1.750**	0.000	0.053
**1.800**	0.000	0.032
**1.850**	0.000	0.018
**1.900**	0.000	0.010
**1.950**	0.000	0.006
**2.000**	0.000	0.003

Gamma Γ: Odds of differential assignment due to unobserved factors.
p_mh+: significance level (assumption: overestimation of exposure effect).
p_mh-: significance level (assumption: overestimation of exposure effect).

Discussion

This study aimed to estimate the impact of chronic malnutrition on the development of EVC in Peruvian children aged 24 to 36 months. After making PSM estimates to match groups of children exposed and not exposed to malnutrition, children exposed to chronic malnutrition were found to be significantly (8%) less likely to show EVC, compared to children of the same age not exposed to malnutrition. These results show that good nutritional status is a key element for adequate ECD. Indeed, according to our analysis, in 2019 almost 14% of Peruvian children aged 24 to 36 months suffered from chronic malnutrition. The presence of this condition could affect proper ECD in Peruvian children, having an impact on language skills and social interaction, which are important for adequate regulation of emotions and behaviors as well as the development of symbolic function (mental representation / mental scheme), and reading and writing skills.

To date, most studies have focused on assessing the impact of malnutrition on subrogated outcomes such as childhood anemia. Our study is one of the first to assess the impact of malnutrition on an objective outcome of the ECD indicator such as EVC, measured by an instrument validated in Peruvian children [19]. The results showed a negative impact of chronic malnutrition on EVC, which in turn may have an impact on the development of other indicators of chronic malnutrition. It was also identified that 40% of Peruvian children between two and three years old would not develop adequate EVC, partly as a result of chronic malnutrition. In this regard, it should be noted that the components of the ECD have intricate mechanisms that interact as the infant matures. For example, safe attachment of children and parents, and stimulation of motor development and gesticulation have been identified as being related to EVC [14–16]. Similarly, adequate nutritional development has a cross-over effect on the development of other indicators of ECD, one of the main being EVC, since it has been reported that chronic malnutrition is associated with poor cognitive development, late school admission and decreased schooling, likely as a consequence of poor EVC development early in life [27]. In this sense, EVC is a problem that can only be effectively addressed by multidisciplinary and multisectoral approaches.

Similarly, a technical report from the INEI in 2018 reported a higher proportion of children aged 9 to 36 months who achieved EVC among residents of urban compared to rural areas (50.5% vs. 47.7%,

respectively), children residing in the jungle vs. the coast and mountain areas (53% vs. 51.9% and 47%, respectively), children of mothers with higher vs. secondary and without education (53% vs. 49.3 and 45%, respectively), boys of the intermediate vs. lower economic quintile (52.3% vs. 46.2%, respectively) and girls vs. boys (54% vs. 45.6%, respectively) [28]. These results demonstrate the presence of gaps according to the sociodemographic characteristics of Peruvian children which must be taken into account by the government in order to target strategies to ensure that a higher proportion of children achieve EVC indicators and to reduce the inequalities in achieving this indicator. EVC is one of the seven components of ECD and its measurement is of interest between 9 and 36 months of age. Indeed, in order to facilitate the work of decision-makers, national studies and technical reports should report chronic malnutrition according to age groups and not aggregated for all children under five years of age (60 months). This would enable focusing on nutritional and complementary strategies for the development of each of the components of the ECD according to specific age groups with potential vulnerability.

Chronic malnutrition continues to be a problem in Peruvian children, with 14% of children between 24 and 36 months suffering from this condition in 2019. This prevalence is above the 9.6% of chronic malnutrition reported in Latin America and the global prevalence of 13.5% in children under five years old in 2017 [29,30]. In addition, it has been described that in the first half of 2019, 33.2% of Peruvian children aged between 24 to 36 months were at risk of chronic malnutrition [31]. Although our country has shown a marked reduction in the prevalence of chronic child malnutrition from 30% in 2005 to 15% in 2015 [32], these results show that efforts are still needed to reduce the prevalence of this condition due to the absence of substantial reductions between 2015 and 2019, and the considerable number of children who remain at risk of chronic malnutrition. Likewise, attention must be paid to the Peruvian Andean region, where according to information from 2018 the lowest proportion of children meet the EVC indicator [28]., with a prevalence of chronic malnutrition of up to 30.1% and a risk of chronic malnutrition in children under five years old of 58.2% [31].

This study has limitations. The use of the secondary database of ENDES for analysis may introduce information biases by the respondents when providing the requested information as these may have provided inaccurate or false information. In addition, this bias may be present due to erroneous introduction of data at the time of data registration by the interviewers. Moreover, since the

sample weights of the survey were not included in our analyses, the results cannot be generalized to the ENDES target population. Despite these limitations, it should be noted that ENDES follows the standardized design and procedures of the Demographic and Health Survey (DHS) program, which includes training of interviewers to reduce the introduction of biases during information collection in order to ensure the quality of the data collected. Similarly, a PSM analysis was used, providing a better balance of covariate measures in groups of children exposed and unexposed to chronic malnutrition with the aim of reducing the risk of confusion bias. This study is also one of the first national reports assessing the association between chronic malnutrition and an objective outcome of the ECD, such as EVC, with representative data.

In conclusion, the presence of chronic malnutrition has a significant negative impact on the propensity to develop EVC in Peruvian children between 24 to 36 months of age. Strategies for reducing malnutrition should be strengthened in population areas identified as vulnerable and should prioritize the components of ECD according to age groups for which their measurement is of interest to complement nutritional strategies with those that directly concern EVC. This would reduce the gaps in EVC among children in Peru with the aim of ensuring adequate ECD and equal opportunities for all Peruvian children.

References

1. 2018 Global Nutrition Report reveals malnutrition is unacceptably high and affects every country in the world, but there is also an unprecedented opportunity to end it. [cited 22 Jun 2020]. Available: https://www.unicef.org/press-releases/2018-global-nutrition-report-reveals-malnutrition-unacceptably-high-and-affects
2. Malnutrition in Children - UNICEF DATA. In: UNICEF DATA [Internet]. [cited 22 Jun 2020]. Available: https://data.unicef.org/topic/nutrition/malnutrition/
3. WHO | Child malnutrition. 2020 [cited 22 Jun 2020]. Available: http://www.who.int/gho/child-malnutrition/en/
4. Black RE, Victora CG, Walker SP, Bhutta ZA, Christian P, de Onis M, et al. Maternal and child undernutrition and overweight in low-income and middle-income countries. Lancet. 2013;382: 427–451.

5. Peru Nutrition Profile - Global Nutrition Report. In: Global Nutrition Report [Internet]. 17 Feb 2020 [cited 22 Jun 2020]. Available: https://globalnutritionreport.org/resources/nutrition-profiles/
6. Workie SB, Mekonen T, Mekonen TC, Fekadu W. Child development and nutritional status in 12-59 months of age in resource limited setting of Ethiopia. J HealthPopulNutr. 2020;39: 6.
7. SISTEMA ODS. [cited 22 Jul 2020]. Available: http://ods.inei.gob.pe/ods/
8. Grantham-McGregor S, Cheung YB, Cueto S, Glewwe P, Richter L, Strupp B, et al. Developmental potential in the first 5 years for children in developing countries. Lancet. 2007;369: 60–70.
9. Lu C, Cuartas J, Fink G, McCoy D, Liu K, Li Z, et al. Inequalities in early childhood care and development in low/middle-income countries: 2010-2018. BMJ GlobHealth. 2020;5: e002314.
10. WHO | Early child development. 2020 [cited 22 Jun 2020]. Available: http://www.who.int/topics/early-child-development/en/
11. Black MM, Walker SP, Fernald LCH, Andersen CT, DiGirolamo AM, Lu C, et al. Early childhood development coming of age: science through the life course. Lancet. 2017;389: 77–90.
12. Liu J, Raine A, Venables PH, Dalais C, Mednick SA. Malnutrition at age 3 years and lower cognitive ability at age 11 years: independence from psychosocial adversity. ArchPediatrAdolescMed. 2003;157: 593–600.
13. de Onis M, Branca F. Childhood stunting: a global perspective. Matern Child Nutr. 2016;12 Suppl 1: 12–26.
14. van IJzendoorn MH, Dijkstra J, Bus AG. Attachment, intelligence, and language: A meta-analysis. Blackwell Publishing; 1995. doi:10.1111/j.1467-9507.1995.tb00055.x
15. Rowe ML, Goldin-Meadow S. Early gesture selectively predicts later language learning. DevSci. 2009;12: 182–187.
16. Longobardi E, Spataro P, Rossi-Arnaud C. The relationship between motor development, gestures and language production in the second year of life: a mediational analysis.InfantBehavDev. 2014;37: 1–4.
17. [No title]. [cited 22 Jun 2020]. Available: https://www.inei.gob.pe/media/MenuRecursivo/publicaciones_digitales/Est/Endes2019/pdf/Lib

ro.pdf

18. [No title]. [cited 12 Aug 2020]. Available: https://apps.who.int/iris/bitstream/handle/10665/63750/WHO_NUT_97.4.pdf;jsessionid=3C2F9C87098F2AF7CA18705293172592?sequence=1
19. [No title]. [cited 10 Aug 2020]. Available: http://evidencia.midis.gob.pe/wp-content/uploads/2019/07/Magaly-Noblega-Validacion-ENDES-19072019.pdf
20. Guo S, Zhao C, Ma Q, Sun H-P, Pan C-W. Prevalence of overweight and malnutrition among ethnic minority children and adolescents in China, 1991-2010. Sci Rep. 2016;6: 37491.
21. Sobrino M, Gutiérrez C, Cunha AJ, Dávila M, Alarcón J. [Child malnutrition in children under 5 years of age in Peru: trends and determinants]. RevPanam Salud Publica. 2014;35: 104–112.
22. Pajuelo Ramírez J, Miranda Cuadros M, Bernui Leo I. Asociación entre altitud de residencia y malnutrición en niños peruanos menores de cinco años. Acta médPeru. 2017;34: 259–265.
23. Flores-Bendezú J, Calderón J, Rojas B, Alarcón-Matutti E, Gutiérrez C. Desnutrición crónica y anemia en niños menores de 5 años de hogares indígenas del Perú: análisis de la encuesta demográfica y de salud familiar 2013. AnFacmed. 2015;76: 135–140.
24. Roy AD. SOME THOUGHTS ON THE DISTRIBUTION OF EARNINGS 1. Oxford EconomicPapers. 1951. pp. 135–146. doi:10.1093/oxfordjournals.oep.a041827
25. Rubin DB. Estimating causal effects of treatments in randomized and nonrandomized studies. Journal of EducationalPsychology. 1974. pp. 688–701. doi:10.1037/h0037350
26. Becker SO, Caliendo M. Sensitivity Analysis for Average Treatment Effects. The Stata Journal: Promoting communications on statistics and Stata. 2007. pp. 71–83. doi:10.1177/1536867x0700700104
27. [No title]. [cited 10 Aug 2020]. Available: http://www.midis.gob.pe/wp-content/uploads/2019/08/DT_Salud_Nutricion_082016v5.pdf
28. [No title]. [cited 10 Aug 2020]. Available: https://cdn.www.gob.pe/uploads/document/file/320706/modulo-DIT-A5-DES-INFANTIL.pdf
29. FAO: Hunger increases in the world and in Latin America and the Caribbean for the third consecutive year. [cited 10 Aug 2020]. Available: http://www.fao.org/americas/noticias/ver/en/c/1152157/#:~:text=Only%20one%20in%20every%20100,of%20age%20in%20the%20region.

30. Roser M, Ritchie H. Hunger and Undernourishment. Our World in Data. 2013 [cited 10 Aug 2020]. Available: https://ourworldindata.org/hunger-and-undernourishment

31. [No title]. [cited 10 Aug 2020]. Available:https://web.ins.gob.pe/sites/default/files/Archivos/cenan/van/informes/2019/Informe%20Gerencial%20SIEN%20HIS%20I%20Semestre%202019-c.pdf

32. Las razones de la reducción de la desnutrición crónica en el Perú*. [cited 10 Aug 2020]. Available: https://ninosdelmilenio.org/novedades/las-razones-de-la-reduccion-de-la-desnutricion-cronica-en-el-peru/

Supporting Information

S1 Table. Database

Figure 1

Click here to access/download;Figure;Figure 1.tif

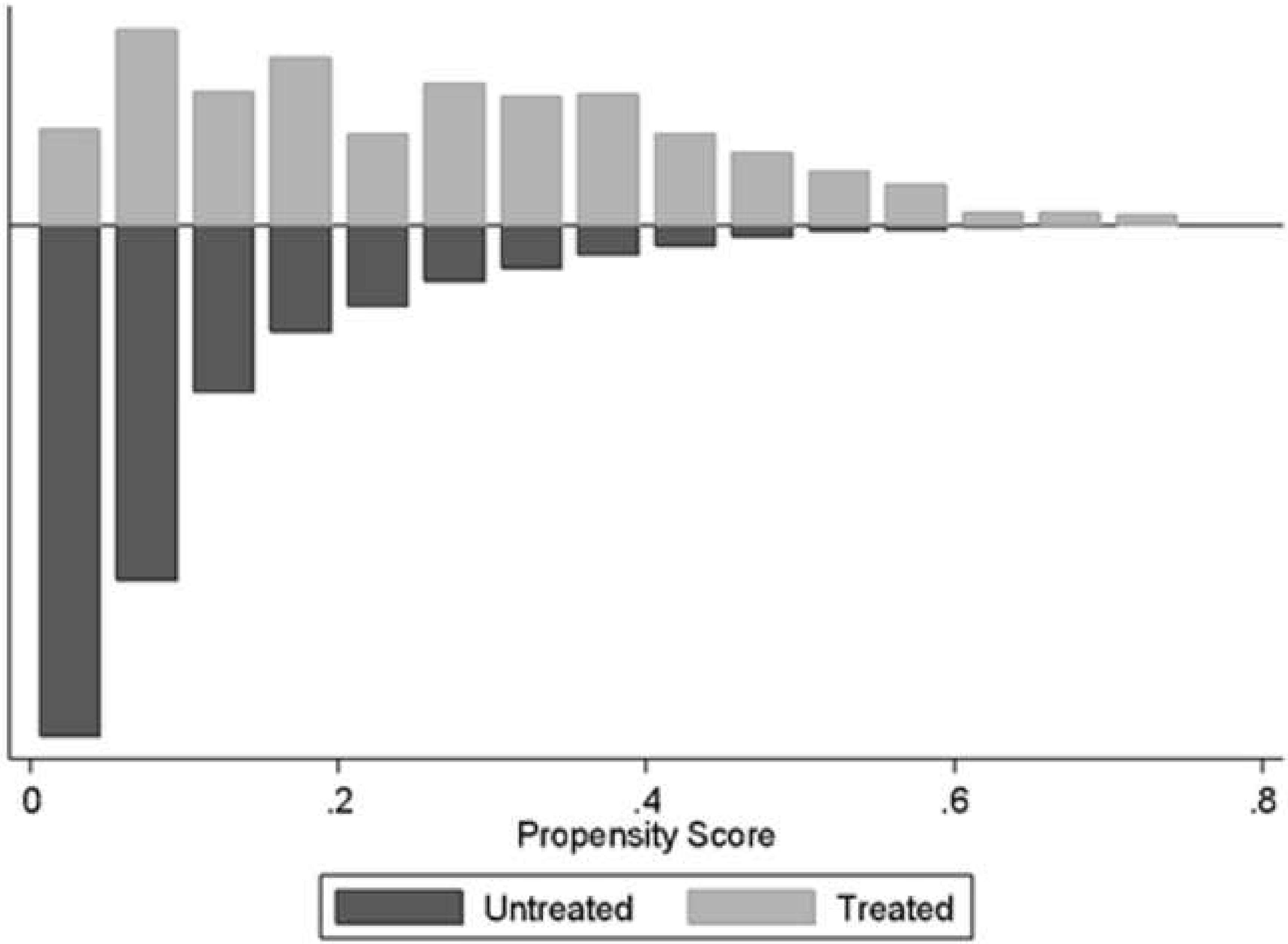

Figure 2

Click here to access/download;Figure;Figure 2.tif

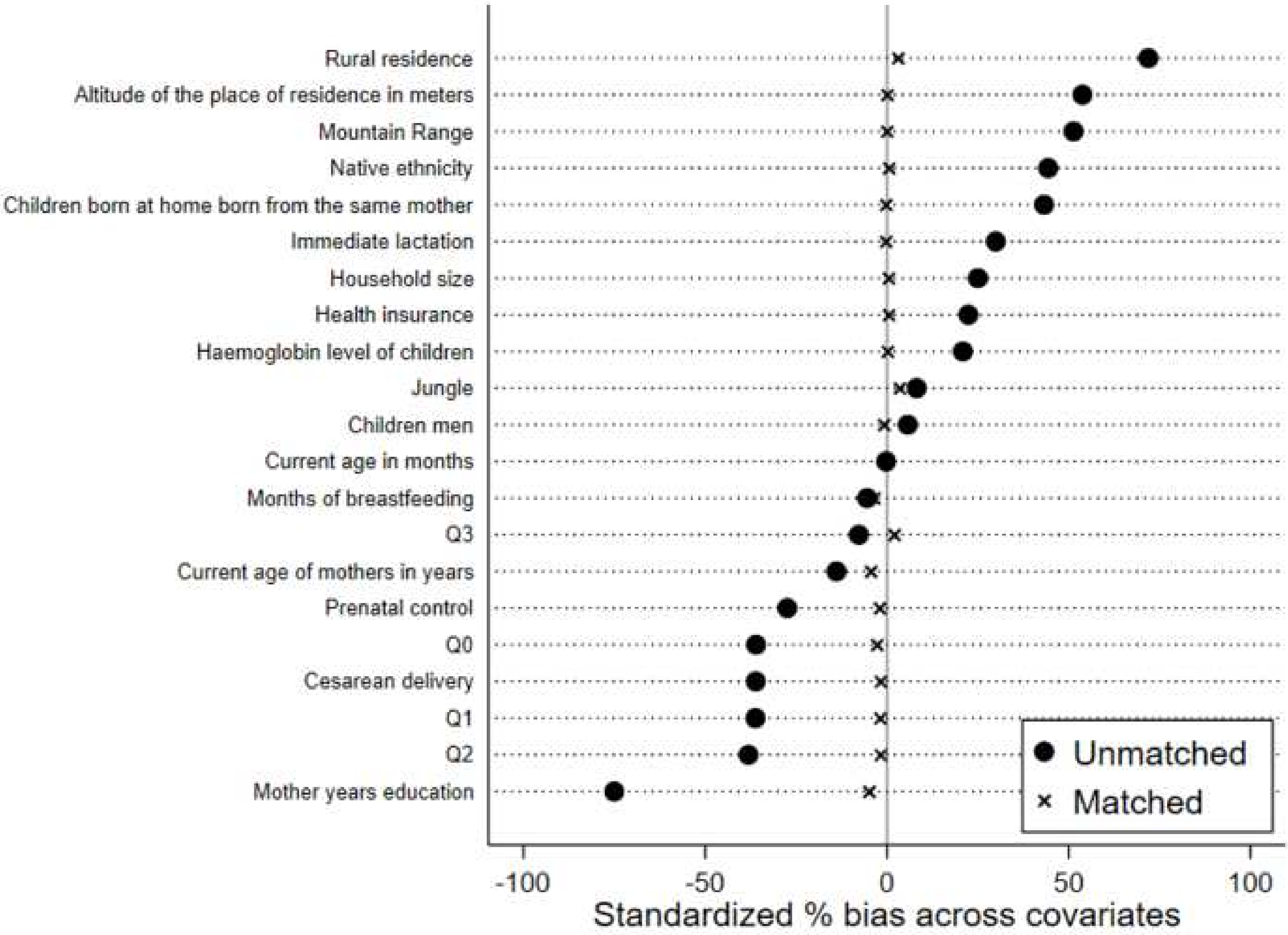

S1 Table. Database

Click here to access/download
Supporting Information
DIT_2019.xls
