# Supplementary material for: Nutritional status and effective verbal communication in Peruvian children: A secondary analysis of the 2019 Demographic and Health Survey
Source: PLoS One. 2021 Feb 18;16(2):e0246542. doi: 10.1371/journal.pone.0246542 (PMC7891687; doi:10.1371/journal.pone.0246542)
Supplement: Attachment — Submitted filename: Review Comments.docx [file pone.0246542.s002.docx]

**In Table 1 & 2:**

The caption should be complete including the which study population and study site. The questions items should be described in the table and mean/SD should be reported separately. Some questions have been labelled Q1 Q2 that should be descried in this table.

**In Table 3**, the caption should include Effective Verbal Communication instead of its abbreviation including the study population, study site and sample size.

**Table 4:** Revise and complete the caption of the table, suggested “sensitivity analysis for estimating the impact of chronic 236 malnutrition on EVC in children aged 24 to 36 months”.

**Study Limitation and its strengths** should be mentioned under a spate heading after end of the research paper.

**The study title** should reflect that it is a secondary data analysis so accordingly study title should be revised.

Kind Regards,

Subhana Akber Khan

Lecturer

Department of Public Health & Nutrition

University of Haripur, KPK.
