# Supplementary material for: Nutritional status and effective verbal communication in Peruvian children: A secondary analysis of the 2019 Demographic and Health Survey
Source: PLoS One. 2021 Feb 18;16(2):e0246542. doi: 10.1371/journal.pone.0246542 (PMC7891687; doi:10.1371/journal.pone.0246542)
Supplement: Attachment — Submitted filename: PLOSone_oct2020.docx [file pone.0246542.s004.docx]

Thank you for the opportunity to read this interesting manuscript that estimates the impact of chronic malnutrition on the development of verbal communication in toddler aged children.

Overall, I think it is a great and well-executed paper. I have a few comments.

**Introduction.**

I really enjoy reading about the Peruvian context and what is being done in Peru in term of supporting the ECD agenda. Here are some minor comments about things that were a little unclear:

1.

“Child malnutrition produces serious metabolic and structural consequences for the individual and has a high social and economic impact [1], mainly in low- and middle-income countries, **where nearly half of the children under five years of age die due to malnutrition [2].”**

This sentence could be improved to be more clear. I read this like you are saying that 50% of all children who are under 5 years in LMIC die due to malnutrition. I suppose that what you mean is that when 50% of the mortality among children <5 years in LMIC is caused by malnutrition?

2.

“This becomes relevant because children **integral development** at an early stage (perceptual, motor, cognitive, language, socio-emotional and self-regulation skills), is highly dependent of an active and healthy life, which cannot be well achieved in the presence of malnutrition [6].”

What do you mean by integral development?

3.

“Early childhood development (ECD) is one of the goals of the Sustainable Development Goals in developing countries [7].:

Can you be more specific as you which goal(s) you refer to? Or do you mean more generally embedded into the SDGs?

4.

“In Peru, the government, led by the Ministry of Development and Social Inclusion, implemented a program aimed at improving ECD. This program included seven areas of development corresponding to a healthy birth, safe attachment, adequate nutritional status, effective verbal communication, walking alone, regulation of emotions and behaviors and symbolic function [8,10].”

This is quite a minor comment, but to me this sounds like the Peruvian government is led by a ministry. What I believe you are trying to say that the ministry led this initiative.

**Methods**

This is a great use of publicly available data and I like for that you mention the specific variable names you have used for replicability although it seems a little unconventional.

5.

I would like a little more information about both the exposure variable and the outcome variable.

For “chronic malnutrition” you simply say that you used WHO’s classification, but what exactly does that mean? Malnutrition is defined as deficient intake of nutrients (macro and micro) but did you use an index of food intake or did you measure an anthropometric proxy like stunting, wasting, middle upper arm circumference? This is important to understand and more clarity is needed. Relatedly, how to you access chronicity? This may be imbedded in a variable like stunted growth which is believed to reflect nutrition over time.

For EVC, you state that it is “performed and answered” by mothers. Exactly what does “performed” mean in this regard? Did you test the child on certain things? How many items does the outcome integrate? Could these maybe be listed in an appendix?

6.

You include a lot of covariates in the analysis and I wonder if all were necessary. A stronger and more specific rationale would be helpful. Are some of these chosen because they may predict child growth status rather than language? E.g., cesarean delivery, prenatal control, "altitude", "immediate lactation"?

**Statistical analysis**

I really appreciate the level of detail provided in the section. I also appreciate the use of three different methods to compare results.

7.

Propensity and match estimation : what is the rationale for the 0.1 cut-off? It seems that are more conservative 0.05 or 0.01 is quite common.

8.

Page 19, line 248 : might you mean under-estimation?

**Results**

No comments

**Discussion:**

10.

You state that 40% of Peruvian children between two and three years old would not develop adequate

EVC, partly as a result of chronic malnutrition. If malnutrition only explains 8% then it does not seem to be the main source of developmental setbacks in communication?

Relatedly, please comment on the extent to which a 8% lower prevalence of EVC is substantial and problematic. This seems like a pretty small effect size. The importance of this effect would also be easier to grasp if you include more information about the outcome variable.

You mention some other variables like attachment and socioeconomic factors that may affect EVC, but these would be accounted for by the matching, correct? So there still seems to be an unexplained gap?

11.

I agree that the PSM approach is a great strength, but you may discuss some of the limitations of PSM methodologies. King and Nielsen (2016) proposed that PSM has serious limitations. Despite their analysis mostly refers to pair matching without replacement, they made interesting points on the fully blocked randomization approach. This is highly debatable, but still a good way to discuss PSM limitations. In particular given the current trends point toward the use of weighting, something used as a secondary approach.
