# Supplementary material for: Nutritional status and effective verbal communication in Peruvian children: A secondary analysis of the 2019 Demographic and Health Survey
Source: PLoS One. 2021 Feb 18;16(2):e0246542. doi: 10.1371/journal.pone.0246542 (PMC7891687; doi:10.1371/journal.pone.0246542)
Supplement: Attachment — Submitted filename: Levantamiento DIT 19012021.docx [file pone.0246542.s006.docx]

Dr. James West

Academic Editor

PLOS ONE

Title: Nutritional status and effective verbal communication in Peruvian children. A secondary analysis of the 2019 Demographic and Health Survey

Dear Editor and Reviewers,

Thank you for your valuable suggestions and remarks. After incorporating your feedback our manuscript has greatly improved. Please find below the point-by-point response to your comment. We hope that the present version of the manuscript is now acceptable for publication, and we look forward to your feedback.

**Comments of Editor:**

**All that remains is including the definition of malnutrition used for this study. Once that's done, it shouldn't need to go back out for review - it can be editorially accepted.**

**Response:** Thank you for the observation. For better understanding we have rewritten the sentence.

Now it reads: “The World Health Organization (WHO) classification (defined by variations of more than 2 standard deviations [SD] of the height for age below the 2006 WHO Child Growth Standards median) was used for the exposure variable "stunting" (HW70) [18] and EVC in children aged between 24 to 36 months (R4).”

**Other changes**

In some cases, “malnutrition” was changed to “stunting” for better understanding of the term.

King regards,

The authors
